# The Architecture of the Golfer's Brain

**DOI:** 10.1371/journal.pone.0004785

**Published:** 2009-03-11

**Authors:** Lutz Jäncke, Susan Koeneke, Ariana Hoppe, Christina Rominger, Jürgen Hänggi

**Affiliations:** 1 Division of Neuropsychology, Institute of Psychology, University of Zurich, Zurich, Switzerland; 2 Department of Biology, Institute of Human Movement Sciences and Sport, ETH Zurich, Zurich, Switzerland; University of Alabama, United States of America

## Abstract

**Background:**

Several recent studies have shown practice-dependent structural alterations in humans. Cross-sectional studies of intensive practice of specific tasks suggest associated long-term structural adaptations. Playing golf at a high level of performance is one of the most demanding sporting activities. In this study, we report the relationship between a particular level of proficiency in playing golf (indicated by golf handicap level) and specific neuroanatomical features.

**Principal Findings:**

Using voxel-based morphometry (VBM) of grey (GM) and white matter (WM) volumes and fractional anisotropy (FA) measures of the fibre tracts, we identified differences between skilled (professional golfers and golfers with an handicap from 1–14) and less-skilled golfers (golfers with an handicap from 15–36 and non-golfer). Larger GM volumes were found in skilled golfers in a fronto-parietal network including premotor and parietal areas. Skilled golfers revealed smaller WM volume and FA values in the vicinity of the corticospinal tract at the level of the internal and external capsule and in the parietal operculum. However, there was no structural difference within the skilled and less-skilled golfer group.

**Conclusion:**

There is no linear relationship between the anatomical findings and handicap level, amount of practice, and practice hours per year. There was however a strong difference between highly-practiced golfers (at least 800–3,000 hours) and those who have practised less or non-golfers without any golfing practise, thus indicating a step-wise structural and not a linear change.

## Introduction

Short and long-term motor and cognitive training is associated with selective and transient neuroanatomical changes in grey and white brain matter in young and older subjects [1]–[4]. The amount of practice is also known to be an important factor in defining the extent of anatomical reorganisations [5]–[8]. But it is not well understood to what extent practice-related neuroanatomical reorganisations are influenced by the level of golfing proficiency.

This study sought to determine whether there are differential neuroanatomical adaptations in golf players with different golf handicaps. The individual level of proficiency of golf players can be objectively and reliably measured on the basis of the “golf handicap”. The golf handicap is a numerical measure ranging from 0 to 54 of a golfer's playing skill, based on the number of strokes actually played in official golf tournaments or on a standard golf course under the control of a professional golf teacher, and adjusted for course difficulty. The smaller the number of strokes played in the player's most recent rounds of golf the lower the handicap. The rules for achieving handicaps are detailed in the official golf rules published by international or national golf associations. A widely used rule for measuring the handicap is detailed by the Council of National Golf Unions (http://www.congu.com/home.asp). To receive a handicap of 0, professional golfers undergo specific tests to demonstrate their exceptional golfing skills. In view of the fact that the golf handicap is strongly associated with the amount of practice [9], we anticipated that the handicap of golfers is associated with the extent of anatomical adaptation.

Playing golf at a high level of achievement places very high demands on motor proficiency. The objective of a golf swing is to produce the intended trajectory of the golf ball by translating the head of the golf club from the top of the backswing to the point of ball contact along a swing plane that is in line with the intended target while ensuring that the face of the club head is perpendicular to this plane. The following five facets of the golf swing illustrate its unique specificity and complexity: (1) The high velocity of the club movement does not facilitate closed-loop control of the movement; thus, the golf swing is a typical example of a ballistic movement. (2) A perfect golf swing is only achieved by coordinating numerous submovements of the arms, hands, legs, feet, shoulders, head, and hips simultaneously and sequentially. (3) Although the different types of golf swings (drives, pitches, chips etc.) have many similar movement characteristics in common, each golf swing variant is dominated by a specific movement pattern that is difficult to incorporate into the basic golf swing. (4) The different golf swings are accomplished with different clubs varying in shaft length, weight, or head size, thus requiring a great variety of tool-body transformations. (5) Finally, the golf swing has to be adapted to an external stimulus (the golf ball) with a high degree of accuracy. Hence, the golf swing is also a typical example of a sensorimotor control task during which a movement has to be aligned according to an external goal.

Frequent practice of the different golf swings is therefore necessary for learning to perform the difficult ballistic movements and for playing golf at a high level of performance. According to many authorities on golf, more than 10000 practice hours are necessary to become an elite and professional golfer. To gain a reasonable handicap of 10–15, at least 5000–10000 practice hours are necessary [9]. Thus, the amount of practice required corresponds with the time invested for practice by professional musicians and music teacher [10]–[12]. According to previous research on experts [1]–[3], [5], [6], [13]–[16], high-intensity golf practice is likely to induce plastic changes in the brain areas associated with the control of the golf swing. A common finding across most skill-acquisition studies is the functional enlargement of the representative brain area that is involved in controlling that particular skill [17]–[21]. In the present study, voxel-based morphometry (VBM) of GM and WM volumes and VBM of fractional anisotropy values (see Methods) were applied to explore whether there are structural brain differences between four otherwise matched groups of subjects who differed in their proficiency status and reported practice intensity. Given that golf swings are ballistic movements aligned according to external stimuli, changes are anticipated in dorsal premotor and parietal cortices, which form part of a brain network known to be essentially involved in the control of sensorimotor tasks, [22], [23]. Anatomical changes of WM are predicted in the corticospinal tracts, which are essentially involved in controlling skilled voluntary movements.

## Methods

### Subjects

We examined 40 healthy males. The sample comprised 10 professional golfers (handicap = 0; professionals: PROs), 10 golfers with a high skill level (handicap>0–14, HCP 0–14), 10 golfers with an intermediate skill level (handicap>15–36, HCP 15–36), and 10 non-golfers (no experience with golf including mini-golf, NOGOLF). The four subgroups were matched for age (confirmed by Kruskal-Wallis nonparametric ANOVA) (see Table 1 for detailed information). Hand preference was determined using the Annett Handedness questionnaire (AHQ) [24]. Applying the criteria of Annett, 80% of the subjects were classified as consistent right-handers (CRH), while the other 20% of subjects were classified as consistent left-handers (CLH). This ratio was the same in all golf proficiency sub-groups. All golfers were recruited by two of the authors (C.R. and L.J.) by personal contact with local golf clubs and via personal contact with the professional golfers. All subjects reported no past or current neurological, psychiatric, or neuropsychological problems, and reported no use of drugs or illegal medication. Subjects were paid for participation. The local ethics committee approved the study and written informed consent was obtained from all participants.

**Table 1:** Subject characteristics of the subjects examined in this study.

Demographic characteristics		Professionals (n = 10)	HCP 1–14 (n = 10)	HCP 15–36 (n = 10)	Non-golfers (n = 10)
**Age (years)**	Mean^a^	30.9	26.6	26.5	25.9
	SD	6.2	8.3	3.4	2.0
	SEM	2.0	2.6	1.1	0.6
	Range	23–42	19–43	22–32	24–31
**Age of commencement (y)**	Mean^b^	13.1	14.5	19.0	n.a.
	SD	5.6	8.4	7.2	n.a.
	SEM	1.8	2.6	2.3	n.a.
	Range	8–27	8–32	9–26	n.a.
**Years playing golf**	Mean^c^	17.8	12.1	7.6	n.a.
	SD	7.9	3.5	6.0	n.a.
	SEM	2.5	1.1	2.0	n.a.
	Range	7–31	7–17	1–16	n.a.
**Hours playing golf per month**	Mean^d^	150.4	31.2	20.4	n.a.
	SD	57.6	29.6	12.6	n.a.
	SEM	18.2	9.3	4.0	n.a.
	Range	56–228	12–88	8–48	n.a.
**Hours playing golf per year**	Mean^d^	1,730	310	141	n.a.
	SD	675	353	106	n.a.
	SEM	213	111	33	n.a.
	Range	672–2,736	84–1,056	32–224	n.a.
**Total hours playing golf**	Mean^d^	27,415	3,207	758	n.a.
	SD	12,542	2,916	737	n.a.
	SEM	3,966	922	233	n.a.
	Range	8,064–46,080	900–8,400	224–2,560	n.a.

**Abbreviations:** HCP, handicap; y, years; n.a., not applicable; SD, standard deviation; SEM, standard error of mean.

a No significant difference between groups confirmed by Kruskal-Wallis nonparametric analysis of variance (p>0.05).

b No significant difference between groups confirmed by analysis of variance (p>0.05).

c Significant difference between groups confirmed by analysis of variance (p<0.01).

d Significant difference between groups confirmed by Kruskal-Wallis nonparametric analysis of variance (p<0.0001).

### Retrospective data

All golfers were interviewed by one of the authors (C.R.) using an in-house developed retrospective questionnaire. With this self-report questionnaire, data on actual *golf handicap*, *age of commencement of playing golf* (in years) and *time spent practicing golf* (in hours and years) were collected. By relating the variable *time spent practicing golf (in hours)* to *years playing golf* we obtained *hours playing golf per year* as an indication of the training impact per month or year. The golfers did not change their practice intensity before or during the period of this study. To ensure that non-golfers had no experience of golf we also asked them whether they played mini-golf, which has some movement features in common with regular golf. In addition, we also asked them whether they have recently or were currently practising any other sport on a regular basis. All non-golfers indicated that they had not played and were not currently playing mini-golf and that they did engage in other sports for recreational purposes no more frequently than once a week.

### Imaging Data Acquisition

Magnetic Resonance Imaging (MRI) scans were acquired on a 3.0 T Philips Intera whole body scanner (Philips Medical Systems, Best, The Netherlands) equipped with a transmit-receive body coil and a commercial eight-element sensitivity encoding (SENSE) head coil array. A volumetric 3D T1-weighted gradient echo sequence scan was obtained with a measured spatial resolution of 1×1×1.5 mm^3^ (acquisition matrix 224×224 pixels, 180 slices) and a reconstructed resolution of 0.86×0.86×0.75 mm^3^ (reconstructed matrix 256×256 pixels, 180 slices). Further imaging parameters were: Field of view FOV = 220×220 mm^2^, echo-time TE = 2.3 ms, repetition-time TR = 20 ms, flip-angle FA = 20°.

Diffusion-weighted spin echo echo-planar (EPI) sequence scans were obtained with a measured spatial resolution of 2.08×2.13×2.0 mm^3^ (acquisition matrix 96×96 pixels, 50 slices) and a reconstructed resolution of 1.56×1.56×2.0 mm^3^ (reconstructed matrix 128×128 pixels, 50 slices). Further imaging parameters were: Field of view FOV = 200×200 mm^2^, echo-time TE = 50 ms, repetition-time TR = 10,166 ms, flip-angle FA = 90°, SENSE factor R = 2.1. Diffusion was measured in 15 non-collinear directions followed by a non-diffusion-weighted volume (reference volume). The b-value was 1,000 s/mm^2^.

### Voxel-Based Morphometry

To investigate local GM and WM volumes we applied the voxel-based morphometry (VBM) algorithm [17] implemented in the VBM5 toolbox (http://dbm.neuro.uni-jena.de/vbm/download/) for the Statistical Parametric Mapping software (SPM5, http://www.fil.ion.ucl.ac.uk/spm/). The morphometric procedure was divided into two steps: Creation of customised a priori maps and actual VBM. The following preprocessing steps were realised. Creation of a priori maps: 1) the coordinate origin of each native MR image was manually set on the anterior commissure. 2) Intensity nonuniform inhomogeneity correction, tissue class segmentation, and spatial normalisation were performed using the unified segmentation approach implemented in SPM5. The canonical a priori maps (ICBM 452 T1-weighted) were used for the creation of customised a priori maps. 3) To enhance tissue class segmentation Hidden Markov Random Field (HMRF) weighting was applied (http://dbm.neuro.uni-jena.de/vbm/markov-random-fields/). 4) For customised a priori map creation the unmodulated, segmented, HMRF weighted GM, WM, and CSF images of all participants were averaged separately and used as a priori maps in the actual VBM. Actual VBM: 5) Steps 1–3 were repeated with the brains of the 40 participants except that the customised a priori maps were used in step 2. 6) To investigate absolute volumes Jacobian modulation was applied to the deformation fields generated during spatial normalisation [18]. 7) The resulting, segmented, Jacobian modulated, and HMRF weighted GM and WM images were smoothed with a Gaussian kernel of FWHM = 9 mm.

Fractional anisotropy (FA) is the most widely used diffusion parameter that represents the anisotropy of water molecule motion. With respect to brain tissue, this motion is stronger along the white matter tracts (axial diffusion) than perpendicular to them (radial diffusion). There is evidence that the FA parameter is sensitive to the coherence and integrity of white matter and to training-induced alterations in the fibre bundles [25], [26]. To analyse interconnectivity of dedicated areas measured by means of fractional anisotropy (FA), we preprocessed the diffusion-weighted images with tools from the Tract-Based Spatial Statistics (TBSS) [27] and the diffusion toolbox (FDT) [28]. This toolbox is part of the FSL software [29] and was used to create fractional anisotropy (FA) maps. The following steps were realised: 1) Head movement and eddy current correction were applied using FDT. 2) Creation of a brain mask of the reference volume (no diffusion) using the brain extraction tool (BET). 3) Tensors were fitted to the data using the DTIFIT tool to generate FA maps. 4) FA maps were scaled and converted. 5) Nonlinear registration of all FA maps into standard space was applied. 6) FA images of the participants were smoothed with a Gaussian kernel of FWHM = 12 mm.

### Statistical analyses

All statistical analyses were performed with the general linear model (GLM) implemented in SPM5. GM and WM voxels with a tissue class probability of lower than 0.2 and FA lower than 0.2 were excluded prior to analyses. Global GM and WM volumes and mean FA were used as a nuisance variable in GM, WM, and FA analysis, respectively. Differences between the four groups as well as the pooled samples (professional and amateur golfers versus novice golfers and non-golfers) were examined using one-way ANCOVAs. In case of significant between-groups differences, subsequent between-group tests were performed. Statistical parametric WM and FA maps were thresholded with a false discovery rate (FDR) of p<0.05 and p<0.1, respectively (corrected for multiple comparisons). Statistical parametric GM maps were thresholded with p<0.0001 uncorrected for multiple comparisons combined with a small volume correction (SVC, p = 0.05) using spheres with a diameter of 40 mm. Based on our hypothesis, we concentrated on anatomical changes in dorsal premotor and parietal cortices, which belong to a brain network known to be essentially involved in the control of sensorimotor tasks. For the WM volumes and the FA values, we concentrated on anatomical changes in the corticospinal tracts, which are essentially involved in controlling skilled voluntary movements.

## Results

### Handicap and retrospective behavioural data

Demographical and behavioural data of the subjects are summarised in Table 1. Because the golf players were selected according to their handicap there were strong differences in handicap between the three golf groups. All PROs had a handicap of 0 while the handicap for the HCP 1–14 group ranged from 1–14 (mean±standard deviation: 7.7±3.5). The handicap for the HCP 15–36 group ranged from 15–36 (28.3±7.9). Subjecting the handicap data to a Kruskal-Wallis nonparametric ANOVA revealed a highly significant between-group difference. In addition, all three groups significantly differed in terms of their handicap (determined with Dunn's multiple comparison test).

The average *time spent practicing golf* (practising driving range, putting, and playing on the golf course) was 27,415±12,542 hours for the PROs, 3,207±2,916 hours for the HCP 1–14 group, and 758±737 hours for the HCP 15–36 group. There was a strong between-group difference for *time spent practicing golf* (Kruskal-Wallis nonparametric ANOVA: Chi^2^ = 22.6, df = 2, p<0.0001). Subsequently performed Dunn's multiple comparisons revealed more *practice time* for the PROs compared to the HCP 1–14 group (p<0.05), and more *practice time* for the PROs compared to the HCP 15–36 group (p<0.001). There was no difference in *practice time* between the HCP 1–14 group and the HCP 15–36 group (p>0.05).

*Mean age of commencement of playing golf* was 13.1±5.6 years for PROs, 14.5±8.4 years for the HCP 1–14 group, 19.0±7.2 years for the HCP 15–36 group. *Mean age of commencement of playing golf* was not significantly different between the three golfer groups (parametric ANOVA: p = 0.17). There was a weak but non-significant linear correlation between *time spent practicing golf* and *age of commencement of playing golf* (r = −0.34, p = 0.07). Therefore, we calculated a linear regression between *time spent practising golf* (as criterion) and *age of commencement of playing golf* (as predictor) and calculated *time spent for practising golf* corrected for the influence of *age of commencement*. These values were subjected to a Kruskal-Wallis nonparametric ANOVA and revealed strong between-group differences for the golf players (Chi^2^ = 18.3, df = 2, p<0.001). The subsequently performed Dunn's multiple comparisons revealed significant differences between the PROs and the HCP 1–14 group (p<0.001) and between the PROs and the HCP 15–36 group (p<0.01). There was no difference between the HCP 1–14 group and the HCP 15–36 group (p>0.05). Thus, the results are the same as for the time spent practicing golf uncorrected for *the age of commencement of playing golf*.

Since we also measured the number of years practising golf we were able to calculate the *number of practice hours per year* in order to have a measure of practising impact. The mean *number of practice hours per year* was strongly and significantly different between the three golf groups (PROs: 1,730±676 hours/year; HCP 1–14: 310±353 hours/year; HCP 15–36: 141±107 hours/year; Kruskal-Wallis nonparametric ANOVA: Chi^2^ = 19.3, df = 2, p<0.001).

### Structural MRI data

In an initial step, one-way ANOVAs showed that the total volumes of GM and WM did not differ between the four groups (PROs, HCP 1–14, HCP 15–36, and NOGOLF). The subsequent VBM analysis comparing the four groups uncovered several brain regions with different GM, WM and FA values. Post-hoc t-tests, however, revealed that there were no differences between PROs and the HCP 1–14 group as well as between the HCP 15–36 and the NOGOLF group. Thus, the between-group variance of this initial ANOVA is primarily explained by the difference between the excellent golfers (PROs+HCP 1–14) vs. the combined further two groups (HCP 15–36+NOGOLF). In the following we will, thus, concentrate on this contrast ((PROs+HCP 1–14) vs. (HCP 15–36+NOGOLF)). The first combined group will be named SKILL 1 while the second group combination will be named SKILL 2 to signal that the common denominator differentiating both groups is the skill level of golf playing.

**Figure 1:**
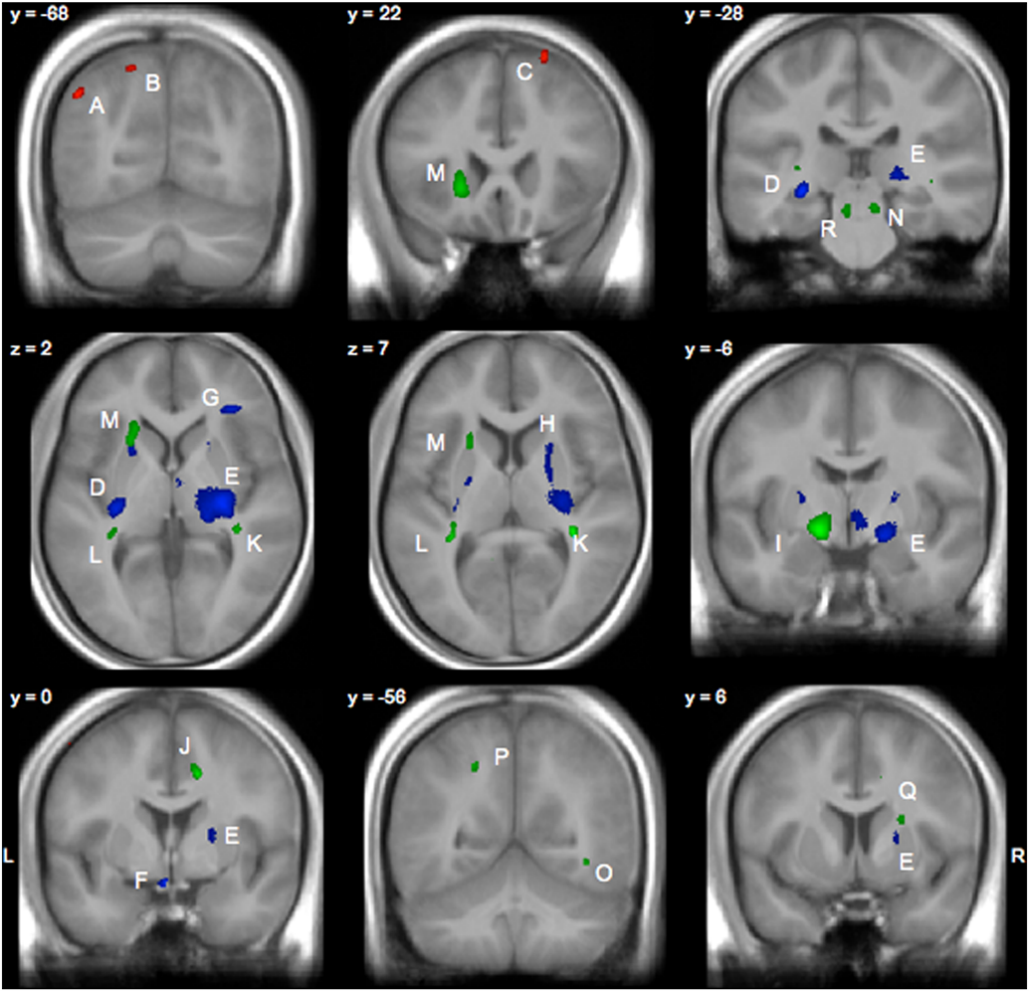
Structural differences in grey matter (GM, in red), white matter (WM, in blue), and fractional anisotropy (FA, in green) between skilled (SKILL1) and less-skilled golfer (SKILL2). In GM regions, skilled golfer showed larger volumes than less-skilled golfer, whereas in WM and FA regions, skilled golfer showed smaller volumes and lower FA than less-skilled golfers. Statistical parametric maps were overlaid on the mean image of the 40 subjects under investigation. Letters refer to the clusters listed in Table 1. y and z are the MNI coordinates.

Subjecting this contrast SKILL 1 vs. SKILL 2 to the VBM analysis of GM volumes revealed strong between-group differences in four brain regions: the right-sided rostral dorsal premotor cortex (pre PMd according to [30]; peak activation: x = 22, y = 22, z = 63; corrected for multiple comparisons FDR = 0.012), the left-sided caudal dorsal premotor cortex (PMd according to [30]; −52, −3, 56; FDR = 0.029), the left-sided posterior parietal cortex in the posterior part of the intraparietal sulcus (−46, −67, 39; FDR = 0.013), and finally in a more medially located region of the posterior part of the parietal cortex (−18, −69, 52; FDR = 0.019). GM volumes in these regions were larger in the SKILL 1 group. There was no brain area with larger GM volumes for the opposite contrast (see also Table 2 and Figure 1).

**Table 2:** Brain regions with significant differences between the SKILL 1 (*PROs* and *HCP1–14*) and SKILL 2 (*HCP 15–36* and *non-golfer*) group with respect to gray matter volume, white matter volume, and fractional anisotropy.

Grey matter volume	Letter in	Hemisphere	MNI			Cluster size	Nonstationarity	t-Value (df = 36)
SKILL 1>SKILL 2	Figure 1		x	y	z	k = 50 voxels	corrected	p<0.0001 (unc.)
Posterior intraparietal sulcus	A	left	−46	−67	39	171	397	5.01
Posterior parietal cortex	B	left	−18	−69	52	62	211	4.72
Dorsal premotor cortex	C	right	22	22	63	137	290	4.94
Caudal premotor cortex	not shown	left	−52	−3	56	50	65	4.72

White matter volume	Letter in	Hemisphere	MNI			Cluster size	Nonstationarity	t-Value (df = 36)
SKILL 2>SKILL 1	Figure 1		x	y	z	k = 50 voxels	corrected	p<0.05 (FDR)
Hippocampus	D	left	−29	−28	−8	1,389	1,560	5.26
External capsule			−31	−22	2			4.76
Fornix			−27	−35	−3			4.32
Corticospinal tract	E	right	28	−18	2	6,290	2,647	5.12
Corticospinal tract			21	−23	−1			4.69
Corticospinal tract			18	−7	−10			4.64
Corpora mamillare	F	left	−3	0	−16	69	298	5.08
Inferior occipitofrontal fascicle	G	right	38	34	−4	557	741	4.79
Inferior occipitofrontal fascicle			28	33	2			3.93
Fronto-orbital cortex	not shown	left	−22	49	−13	173	337	4.48
Putamen	not shown	left	−22	11	−1	169	234	4.46
Inferior occipitofrontal fascicle	not shown	left	−32	−17	18	106	60	4.01
Anterior limb of internal capsule	H	right	21	1	7	446	130	3.92
Anterior thalamic radiation	not shown	left	−6	−13	−4	123	61	3.91

Fractional anisotropy	Letter in	Hemisphere	MNI			Cluster size	Nonstationarity	t-Value (df = 36)
SKILL 2>SKILL 1	Figure 1		x	y	z	k = 50 voxels	corrected	p<0.10 (FDR)
Corticospinal tract	I	left	−14	−6	−8	875	795	5.10
Supplementary motor area	J	right	14	0	40	208	596	4.69
Posterior limb of internal capsule	K	right	35	−35	8	383	522	4.86
Posterior limb of internal capsule	L	left	−33	−38	6	309	589	4.53
Corpus callosum	M	left	−15	32	−9	1,739	1,145	4.53
Inferior occipitofrontal fascicle		left	−21	24	−3			4.49
Inferior occipitofrontal fascicle		left	−23	16	3			4.00
Posterior cingulum	not shown	left	−11	−51	14	207	323	4.54
Corticospinal tract	N	right	9	−26	−17	312	239	4.22
Superior frontal gyrus	not shown	left	−16	28	47	51	53	4.16
Cingulum	not shown	left	−10	−22	36	78	193	3.95
Optic radiation, inferior longitudinal fascicle	O	right	37	−56	−7	68	120	3.82
Anterior intraparietal sulcus	P	left	−21	−56	43	80	84	3.81
Internal capsule	Q	right	24	6	17	90	126	3.70
Corticospinal tract	R	left	−6	−27	−18	126	72	3.66

With respect to the analysis of WM volume, we identified the opposite effect (SKILL 2>SKILL 1) in the vicinity of the corticospinal tract at the level of the internal and external capsule and in the parietal operculum. There were additional clusters of decreased WM volume in the orbito-frontal cortex (left side) and in the corpora mamillare.

A slightly different picture was found for the FA data. In line with the WM results, FA values in the vicinity of the internal capsule were smaller for the SKILL 1 group compared to the SKILL 2 group. However, the corresponding clusters were located at the anterior and posterior limbs of the internal capsule and not in the middle parts comprising the pyramidal tracts coming from the motor cortex. Additionally, there were decreased FA values for the SKILL 1 group in the posterior part of the corpus callosum, in the retrolenticular part of the internal capsule (forming the optic radiation), in the anterior corona radiata, in the inferior longitudinal fascicle, in the external capsule, and in the afferent fibers in the pons (probably representing the lateral lemnicus) (see Table 2 and Figure 1).

## Discussion

The findings of this study suggest a pattern of neuroanatomical differences between the SKILL 1 (comprising the PROS and the HCP 1–14 group) and SKILL 2 groups (comprising the HCP 15–36 and NOGOLF) in brain regions involved in the control of sensorimotor and cognitive processes. Although anatomical differences between the SKILL 1 and SKILL 2 groups were found, no differences were evident either between the excellent golfers (PROs and HCP 1–14) or between the intermediate golfers (HCP 15–36 and NOGOLF). The extent of anatomical adaptations found does not therefore linearly correlate with the level of golfing skill as measured by handicap and hours of practice. These data nevertheless support a categorical differentiation of anatomical organisation between golfers with low (PROs and HCP 1–14) and high handicap levels (HCP 15–36).

Considering that the golf handicap strongly depends on the amount of accumulated practice (*total practice time in years or months*), the current finding supports the idea that neuroanatomical changes are induced by intensive golf practice. But the evident lack of differences between PROs and the HCP 1–14 group, even though the former practiced approximately 8 times as much as the latter, may indicate that the anatomical differences were induced predominantly in the early phases of golfing practice (e.g. within the first 800 to 3,000 practising hours). Even if we use *hours of practice per year* as an indicator of practice intensity instead of the *total practice time*, the anatomical differences cannot be explained entirely by this variable. The yearly practice time of professional golfers is approximately 5–6 times greater than that of HCP 1–14 golfers, while HCP 1–14 golfers practise only twice as much as HCP 15–36 golfers. There is accordingly a greater difference in self-reported practice intensity between PROs and HCP 1–14 golfers than between HCP 1–14 and HCP 15–36 golfers. As an explanatory variable, the *age of commencement of playing golf* does not help any further because there are no differences in this variable between the three golf groups of this study. These data are consistent with the view that the anatomical changes might have occurred at some point after the first 800–3000 practice hours or after a practice impact of more than 310 practice hours per year. In other words, anatomical changes may be induced by decreasing the golf handicap in early training phases to a handicap of approximately 15, whereas further practice, which is evidently necessary to achieve the proficiency of an elite golfer (associated with an average total of 27,000 practicing hours or 1,730 practise hours per year in this study), does not contribute any further to neuroanatomical reorganisation. The idea of qualitative steps in training-induced neuroanatomical change is consistent with the recent finding of a longitudinal study in which healthy subjects were required to learn a three-ball cascade-juggling task. The ability to learn the juggling task was correlated with an increase in GM, whereas further training-induced improvement over time did not alter brain structure [4]. Possible further adaptations with increasing practice time might take place at a functional rather than neuroanatomical level.

Most of the brain areas in which an increase in the GM volumes of the SKILL 1 group were found are located in the left hemisphere and comprise the so-called PMd proper and parts of the posterior parietal cortex (PPC). The core function of PMd proper is spatial information processing in the context of movement generation and preparation achieved in interaction with the PPC [30]. Our finding of GM increases in the left PMd proper and PPC might be connected with the fact that most golfers of our study (80%) are strongly right-handed. It is known that the left-sided sensorimotor system is more strongly involved in the control of complex bimanual movements in right-handers [31], [32]. Increased GM in golfers of the SKILL 1 group was also found in the right hemisphere in the more rostrally located pre-PMd. The pre-PMd has been shown to be more closely related to cognitive aspects of movement control. For example, activation increases in the pre-PMd have been shown during sensory-motor associations tasks, conditional motor tasks, during presentation of visual cues related to specific movements, and during complex working memory, or movement imagination tasks [30], [33].

The preceding task-related psychological processes are typically engaged by golfers before and during the golf swing, in that the golfer has to prepare and generate the appropriate movement on the basis of careful analysis of spatial information. In addition, most golfers imagine the swing they intend to execute, and they do this on the basis of careful analysis of available visual cues.

The finding of focal training-dependent GM increases is in line with several recent cross-sectional imaging studies that show differences in GM volume between groups with different levels of skill. These GM adaptations are evident in those brain regions that are involved in controlling a particular skill [1]–[3], [7], [13], [34]. Several recent longitudinal studies have reported changes in GM densities in brain areas involved in controlling the task for the subject was trained over the course of several weeks or months [1]–[4], [35]. Our data are therefore interpreted in line with these recent experiments as reflecting experience-dependent alterations of GM in specific brain areas. However, the microscopic and macroscopic basis of such training-induced increases in GM volumes measured using T1-weighted magnetic resonance imaging (MRI) is still not very clear. The macroscopic changes may be attributable to an increase in cell size, genesis of glial or even neural cells, or changes in spine density, blood flow, or interstitial fluid [1]–[4], [35]. Further experiments are needed to compare imaging results with histological data for identification of the structural basis of these training-dependent structural changes in human brains, both at the microscopic and macroscopic levels. This would provide the basis for more substantial interpretation of findings obtained with “coarse” methods such as MRI. One example of a paradigm would be to study mice or rats before and after acquiring specific skills and to examine whether any histological changes in their brains correlate with anatomical features in the brain of rats and mice measured with structural MRI.

Decreased WM volume and FA values in several brain structures such as the corticospinal tract, internal and external capsule, and inferior occipitofrontal fascicle have been found in skilled compared with less-skilled golfers. However, the locations of the clusters obtained from the analyses of WM and FA are not perfectly matched. One reason may be that the VBM method has a reduced sensitivity for the detection of WM volume differences. In the opinion of the authors, the findings pertaining to the WM volume should be interpreted more cautiously than the findings for GM volumes and FA.

Most of the focal decreases of WM volume in SKILL 1 group of excellent golfers are found bilaterally in the corticospinal tract in the vicinity of the putamen and pallidum (at the level of the internal and/or external capsule), the external capsule, and the parietal operculum. There are also some clusters of decreased WM volumes in the orbitofrontal cortex (left side) and the corpora mamillare. Although clusters of decreased FA are also found in the vicinity of the internal capsule, the peaks are located either at the anterior or posterior part of the internal capsule and not at the level of the motor tracts, such as the clusters obtained from the WM-volume analysis. The anterior part of the internal capsule is part of the so-called thalamocortical tract, containing fibers that run from the thalamus to the frontal lobe, fibers that connect the lentiform and caudate nuclei, fibers that connect the cortex with the corpus striatum, and fibers passing from the frontal lobe through the medial fifth of the base of the cerebral peduncle to the nuclei pontis. This system is principally involved in regulating emotion, attention, and basic movement processes [36], [37].

Why excellent golfers (SKILL 1) show decreased WM volumes and FA values in these brain areas is difficult to explain on the basis of current knowledge about structure-function relationship and WM architecture. It is conceivable that the identified fibres in this group are not as strongly involved in controlling the golf swing, thus causing the reduction of WM. It is also possible that skilled golfers are more proficient in using optimised control strategies involved in controlling the golf swing, thereby reducing the reliance on the WM system.

Executing a golf swing involves the repeated use of manifold movements of the entire body. These movements have to be conducted in a highly automated, precise, and well-timed fashion to accomplish an efficient golf swing. The highly frequent activation of the ‘automated movement’ circuits (controlled by the striatum and the cerebellum) by the professional golfers is hypothesised to lead to a change in WM anatomy. The differences observed in the corticospinal tract (corona radiata and internal capsule) appear to reflect the presence of a larger repertoire of automated motor programs. The current findings of differences in WM structure therefore hint at underlying differences in the extent of use and/or functional efficiency of the primary motor areas. Although these findings cannot be explained satisfactorily, it should be emphasised that at least two recent DTI studies have also reported significantly reduced FA in the corona radiata and the internal capsule, bilaterally, in a group of highly skilled professional musicians [14], [38].

In summary, we report the relationship between a particular level of golfing proficiency and specific neuroanatomical features identified in cortical grey matter and white matter architecture. In line with recent papers, we hypothesise that these reorganisations are due to the different intensities of practice. Alternatively, extremes or particular patterns of normal anatomical variability may foster the development of extraordinary abilities. If this is the case, a special anatomy would be a prerequisite for advanced skill acquisition rather than the consequence of it. Should these structural differences be in fact innate, individuals exhibiting such differences in brain anatomy might be drawn to becoming professional golfers by virtue of the greater ease with which the golf swing is mastered. Although self-selection for golfers (or any kind of specialised behaviour) cannot be completely ruled out, several studies strongly support the hypothesis that the human brain can be shaped by experience. For example, a wealth of data from animal experiments examining structural brain effects of skill acquisition and long-term motor training support the proposal that volumetric structural differences are caused by training. However, only future experiments can determine the relative contribution of predisposition and practice.

## References

[1] Boyke J, Driemeyer J, Gaser C, Buchel C, May A (2008). Training-Induced Brain Structure Changes in the Elderly.. Journal of Neuroscience.

[2] Draganski B, Gaser C, Busch V, Schuierer G, Bogdahn U (2004). Neuroplasticity: changes in grey matter induced by training.. Nature.

[3] Draganski B, Gaser C, Kempermann G, Kuhn HG, Winkler J (2006). Temporal and spatial dynamics of brain structure changes during extensive learning.. J Neurosc.

[4] Driemeyer J, Boyke J, Gaser C, Büchel C, May A (2008). Changes in gray matter induced by learning-revisited.. PLoS ONE.

[5] Aydin K, Ucar A, Oguz KK, Okur OO, Agayev A (2007). Increased Gray Matter Density in the Parietal Cortex of Mathematicians: A Voxel-Based Morphometry Study.. AJNR Am J Neuroradiol.

[6] Gaser C, Schlaug G (2003). Brain structures differ between musicians and non-musicians.. The J Neurosci.

[7] Cannonieri GC, Bonilha L, Fernandes PT, Cendes F, Li LM (2007). Practice and perfect: length of training and structural brain changes in experienced typists.. Neuroreport.

[8] Maguire EA, Gadian DG, Johnsrude IS, Good CD, Ashburner J (2000). Navigation-related structural change in the hippocampi of taxi drivers.. PNAS.

[9] Brunton H (2007). The Development of Expertise for Elite Competitive Golfers and the Related Probability of Advancing to the PGA Tour–Key Information for Athletes, Parents, Coaches, Golf Professionals and Administrators.. http://cpga.com/UserFiles/CPGAMastersPaper-FINALEDITED-REVISED%20NOV(1).pdf.

[10] Ericsson KA, Krampe RT, Heizmann S (1993). Can we create gifted people?. Ciba Found Symp.

[11] Ericsson KA, Lehmann AC (1996). Expert and exceptional performance: evidence of maximal adaptation to task constraints.. Annu Rev Psychol.

[12] Münte TF, Altenmüller E, Jancke L (2002). The musician's brain as a model of neuroplasticity.. Nature Rev Neurosci.

[13] Mechelli A, Crinion JT, Noppeney U, O'Doherty J, Ashburner J (2004). Neurolinguistics: structural plasticity in the bilingual brain.. Nature.

[14] Schmithorst VJ, Wilke M (2002). Differences in white matter architecture between musicians and non-musicians: a diffusion tensor imaging study.. Neurosci Lett.

[15] Sluming V, Barrick T, Howard M, Cezayirli E, Mayes A (2002). Voxel-based morphometry reveals increased gray matter density in Broca's area in male symphony orchestra musicians.. Neuroimage.

[16] Lee H, Devlin JT, Shakeshaft C, Stewart LH, Brennan A (2007). Anatomical traces of vocabulary acquisition in the adolescent brain.. J Neurosci.

[17] Karni A, Meyer G, Jezzard P, Adams MM, Turner R (1995). Functional MRI evidence for adult motor cortex plasticity during motor skill learning.. Nature.

[18] Koeneke S, Lutz K, Herwig U, Ziemann U, Jancke L (2006). Extensive training of elementary finger tapping movements changes the pattern of motor cortex excitability.. Exp Brain Res.

[19] Pascual-Leone A, Nguyet D, Cohen LG, Brasil-Neto JP, Cammarota A (1995). Modulation of muscle responses evoked by transcranial magnetic stimulation during the acquisition of new fine motor skills.. J Neurophysiol.

[20] Schlaug G, Knorr U, Seitz R (1994). Inter-subject variability of cerebral activations in acquiring a motor skill: a study with positron emission tomography.. Exp Brain Res.

[21] Toni I, Krams M, Turner R, Passingham RE (1998). The time course of changes during motor sequence learning: a whole-brain fMRI study.. Neuroimage.

[22] Jancke L, Loose R, Lutz K, Specht K, Shah NJ (2000). Cortical activations during paced finger-tapping applying visual and auditory pacing stimuli.. Brain Res Cogn Brain Res.

[23] Mars RB, Hulstijn W, Toni I (2008). Selection, preparation, and monitoring: current approaches to studying the neural control of action.. Cortex.

[24] Annett M (1970). A classification of hand preference by association analysis.. Br J Psychol.

[25] Assaf Y, Pasternak O (2008). Diffusion tensor imaging (DTI)-based white matter mapping in brain research: a review.. J Mol Neurosci.

[26] Mori S, Zhang J (2006). Principles of diffusion tensor imaging and its applications to basic neuroscience research.. Neuron.

[27] Smith SM, Jenkinson M, Johansen-Berg H, Rueckert D, Nichols TE (2006). Tract-based spatial statistics: voxelwise analysis of multi-subject diffusion data.. Neuroimage.

[28] Behrens TEJ, Woolrich MW, Jenkinson M, Johansen-Berg H, Nunes RG (2003). Characterization and propagation of uncertainty in diffusion-weighted MR imaging.. Magn Reson Med.

[29] Smith SM, Jenkinson M, Woolrich MW, Beckmann CF, Behrens TE (2004). Advances in functional and structural MR image analysis and implementation as FSL.. Neuroimage.

[30] Picard N, Strick PL (2001). Imaging the premotor areas.. Curr Opin Neurobiol.

[31] Viviani P, Perani D, Grassi F, Bettinardi V, Fazio F (1998). Hemispheric asymmetries and bimanual asynchrony in left- and right-handers.. Exp Brain Res.

[32] Schulze K, Lüders E, Jancke L (2002). Intermanual transfer in a simple motor task.. Cortex.

[33] Abe M, Hanakawa T, Takayama Y, Kuroki C, Ogawa S (2007). Functional coupling of human prefrontal and premotor areas during cognitive manipulation.. J Neurosci.

[34] Golestani N, Paus T, Zatorre RJ (2002). Anatomical correlates of learning novel speech sounds.. Neuron.

[35] May A, Hajak G, Ganssbauer S, Steffens T, Langguth B (2007). Structural brain alterations following 5 days of intervention: dynamic aspects of neuroplasticity.. Cer Cortex.

[36] Kiehl KA (2006). A cognitive neuroscience perspective on psychopathy: evidence for paralimbic system dysfunction.. Psychiatry Res.

[37] Sudhyadhom A, Bova FJ, Foote KD, Rosado CA, Kirsch-Darrow L (2007). Limbic, associative, and motor territories within the targets for deep brain stimulation: potential clinical implications.. Curr Neurol Neurosci Rep.

[38] Bengtsson SL, Nagy Z, Skare S, Forsman L, Forssberg H (2005). Extensive piano practicing has regionally specific effects on white matter development.. Nature Neurosci.

